# Finding Community Modules of Brain Networks Based on PSO with Uniform Design

**DOI:** 10.1155/2019/4979582

**Published:** 2019-11-17

**Authors:** Jie Zhang, Lingkai Tang, Bo Liao, Xiaoshu Zhu, Fang-Xiang Wu

**Affiliations:** ^1^School of Computer Science and Engineering, Guangxi Colleges and Universities Key Lab of Complex System Optimization and Big Data Processing, Yulin Normal University, Yulin 537000, Guangxi, China; ^2^Division of Biomedical Engineering and Department of Mechanical Engineering, University of Saskatchewan, Sakatoon S7N5A9, Saskatchewan, Canada

## Abstract

The brain has the most complex structures and functions in living organisms, and brain networks can provide us an effective way for brain function analysis and brain disease detection. In brain networks, there exist some important neural unit modules, which contain many meaningful biological insights. It is appealing to find the neural unit modules and obtain their affiliations. In this study, we present a novel method by integrating the uniform design into the particle swarm optimization to find community modules of brain networks, abbreviated as UPSO. The difference between UPSO and the existing ones lies in that UPSO is presented first for detecting community modules. Several brain networks generated from functional MRI for studying autism are used to verify the proposed algorithm. Experimental results obtained on these brain networks demonstrate that UPSO can find community modules efficiently and outperforms the other competing methods in terms of modularity and conductance. Additionally, the comparison of UPSO and PSO also shows that the uniform design plays an important role in improving the performance of UPSO.

## 1. Introduction

Graph theory is a very helpful mathematical tool in the field of brain network analysis [1–3]. A brain can be represented as a modular network [4, 5], which is composed of some important neural unit modules. They can provide us rich and useful information and exhibit small-world properties of brain networks [6]. These modules are known as community modules. In brain networks, each vertex denotes a region of interest (ROI) [7], and each edge and its weight represent the connectivity and its strength, respectively [8–10].

Community detection methods are frequently used to find community modules. Girvan and Newman proposed the concept of modularity [11–13], which is the widely used and best known metric. A larger modularity represents a better community partition. Modularity-based community detection methods find the best community modules by seeking the maximum modularity. Namely, when the modularity is maximal, the methods terminate. Therefore, community detection methods can be addressed by means of optimization methods. The FastQ [14] community detection method uses a greedy optimization to maximize modularity. It repeatedly joins communities together in pairs by choosing the join that results in the maximum alteration of modularity in each step. Danon et al.'s [15] community detection method is a modification of FastQ, in which the communities of different sizes are treated equally. The Louvain [16, 17] community detection method firstly calculates the gain of modularity by exchanging a node to its neighbor nodes. Then, the neighbor node obtaining the maximum gain replaces the node.

Particle swarm optimization (PSO) [18–20], as one of the swarm intelligent optimization algorithms, was first put forward by Eberhart and Kennedy [21, 22]. It simulates the foraging process of birds. Each bird (particle) may search the feasible solution space individually and share its individual optimal information to the other bird (particle). The swarm can obtain the global optimal solution by comparing the best solutions of all birds (particles) in the swarm. PSO can obtain the optimal solution quickly. However, it has the drawback of premature convergence [23].

The uniform design belongs to the category of the pseudo-Monte Carlo method. It can generate the solutions scattered uniformly over the vector space, and the solutions are independent of each other [24–26]. The uniform design can be applied to many problems, including bio-inspired intelligent optimizations. Zhang et al. [27] combined the uniform design and artificial bee colony to find the community of brain networks. Zhang et al. [26] introduced the uniform design into association rule mining and presented a multiobjective association rule mining algorithm based on the attribute index and the uniform design. Leung and Wang [24] integrated the uniform design and the multiobjective genetic algorithm to obtain the Pareto optimal solutions uniformly over the Pareto frontier. Zhu et al. [28] combined the uniform design and PAM to find the Pareto optimal solutions of the multiobjective particle swarm optimization. Dai and Wang [29] presented a new decomposition-based evolutionary algorithm with the uniform design. Liu et al. [30] proposed a hybrid genetic algorithm based on the variable grouping and the uniform design for global optimization problems. Tan et al. [31] adopted the uniform design to set the aggregation coefficient vectors of the subproblems and proposed the uniform design multiobjective evolutionary algorithm based on decomposition. Feng et al. [32] presented a uniform dynamic programming to alleviate the dimensionality problem of dynamic programming by means of introducing a uniform dynamic to dynamic programming.

There are only a few reports on community detection in brain networks in the literature. Liao et al. [33] utilized U-Net-based deep convolutional networks to identify and segment the brain tumor. Williams et al. [34] utilized both Louvain [16] and Infomap [35] community detection algorithms to identify modules in noisy or incomplete brain networks. Zhang et al. [27] utilized the artificial bee colony with the uniform design to detect community modules of brain networks. Wang et al. [36] used the multiview nonnegative matrix factorization to detect modules in multiple biological networks.

This study presents a novel method to find community modules of brain networks by integrating PSO with the uniform design. PSO is used to maximize modularity, while the uniform design is used to alleviate premature convergence of PSO by generating sampled points scattered evenly over the vector space.

The rest of this study is organized as follows: Section 2 describes the preliminaries of UPSO.  Section 3 introduces two evaluation metrics. The dataset and the preprocessing method to be used are described in Section 4. The details of UPSO are shown in Section 5. The comparison between UPSO and several competing algorithms is illustrated in Section 6. The conclusion and future work are described in Section 7.

## 2. Preliminaries

In this section, we describe PSO and the uniform design.

### 2.1. Particle Swarm Optimization

In a *d*-dimensional search space, the position and velocity of the *i*-th particle are, respectively, represented as *x*_*i*_=[*x*_*i*,1_, *x*_*i*,2_,…, *x*_*i*,*d*_] and *s*_*i*_=[*s*_*i*,1_, *s*_*i*,2_,…, *s*_*i*,*d*_], where *i*=1,2,…, *N*_pop_, in which *N*_pop_ denotes the population size. The optimal solution of the *i*-th particle is called the individual optimum, while the optimal solution of the whole swarm is called the global optimum. They, respectively, are denoted as *P*_best_*i*__=[*p*_*i*,1_, *p*_*i*,2_,…, *p*_*i*,*d*_] and *G*_best_=[*p*_*g*,1_, *p*_*g*,2_,…, *p*_*g*,*d*_]. The following formulas are utilized to update the velocity and position of each particle in the swarm [21, 22], respectively:

(1) $$\begin{array}{c} s_{i}\left(t+1\right)=\omega \cdot s_{i}\left(t\right)+c_{1}\cdot r_{1}\left(P_{{\text{best}}_{i}}\left(t\right)-x_{i}\left(t\right)\right) \end{array}$$

(2) $$\begin{array}{c} x_{i}\left(t+1\right)=x_{i}\left(t\right)+s_{i}\left(t+1\right), \end{array}$$

where *i*=1,2,…, *N*_pop_; *ω* is called the inertia weight coefficient reflecting the ability to track the previous speed; *c*_1_ and *c*_2_ are called the acceleration coefficients of the individual and the global optimum, respectively, and are commonly set as 2; and *r*_1_ and *r*_2_ are two random numbers distributed uniformly in (0, 1).

From the theoretical analysis of a PSO algorithm, the trajectory of a particle *x*_*i*_ converges to the mean of *P*_best_*i*__ and *G*_best_. Whenever the particle converges, it “flies” to the individual best position and the global best position [37]. According to formulas (1) and (2), the individual optimum position of each particle gradually moves closer to the global optimum position. Therefore, all the particles may converge to the global optimum position.

### 2.2. Uniform Design

The uniform design is an experimental design method. Its main objective is to sample a small set of points from a given set of points such that the sampled points are uniformly scattered.

Let *n* be the number of factors and *q* be the number of levels per factor. When *n* and *q* are given, the uniform design selects *q* combination from all *q*^*n*^ possible combinations such that these combinations are scattered uniformly over the space of all possible combinations. The selected *q* combinations are expressed in a uniform array *U*(*n*, *q*)=[*U*_*l*_1_,*l*_2__]_*q*×*n*_, where *U*_*l*_1_,*l*_2__ is the level of the *l*_2_-th factor in the *l*_1_-th combination and can be calculated by the following formula [24–26, 28, 29, 38]:

(3) $$\begin{array}{c} U_{l_{1},l_{2}}=\left(l_{1}\cdot \sigma^{l_{2}-1}\mathrm{mod}q\right)+1, \end{array}$$

where *σ* is a parameter given in Table 1.

Based on the uniform design, a crossover operator is as follows [24]. It quantizes the solution space defined by two parents into a finite number of points and then applies the uniform design to select a small sample of uniformly scattered points as the potential offspring.

Consider two parents *x*_1_=(*x*_1,1_, *x*_1,2_,…, *x*_1,*d*_) and *x*_2_=(*x*_2,1_, *x*_2,2_,…, *x*_2,*d*_). The minimal and maximal values of each dimension for *x*_1_ and *x*_2_ can generate a novel solution space [*l*_parent_, *u*_parent_], denoted as follows:

(4) $$\begin{array}{c} \left\{\begin{array}{c} l_{\text{parent}}=\left[\min\left(x_{1,1},x_{2,1}\right),\min\left(x_{1,2},x_{2,2}\right),\ldots ,\min\left(x_{1,d},x_{2,d}\right)\right],\\ u_{\text{parent}}=\left[\max\left(x_{1,1},x_{2,1}\right),\max\left(x_{1,2},x_{2,2}\right),\ldots ,\max\left(x_{1,d},x_{2,d}\right)\right]. \end{array}\right. \end{array}$$

Each domain of [*l*_parent_, *u*_parent_] is quantized into *Q*_1_ levels *β*_*i*,1_, *β*_*i*,2_,…, *β*_*i*,*Q*_1__, where *Q*_1_ is a predefined prime number and *β*_*i*,*j*_ is given as follows:

(5) $$\begin{array}{c} \beta_{i,j}=\left\{\begin{array}{cc} \min\left(x_{1,i},x_{2,i}\right), & j=1,\\ \min\left(x_{1,i},x_{2,i}\right)+\left(j-1\right)\left(\frac{\left|x_{1,i}-x_{2,i}\right|}{Q_{1}-1}\right), & 2\leq j\leq Q_{1}-1,\\ \max\left(x_{1,i},x_{2,i}\right), & j=Q_{1}. \end{array}\right. \end{array}$$

Then, the uniform design is applied to select a sample point as the potential offspring. The crossover operator of two parents *x*_1_ and *x*_2_ can acquire *Q*_1_ offsprings, which are scattered evenly over the vector space spanned by *x*_1_ and *x*_2_. More details of the algorithm can be obtained from references [24, 25].

## 3. Evaluation Metrics

There exist many evaluation metrics for community modules of complex brain networks. In this study, we adopt the following metrics.

### 3.1. Modularity [27]

The modularity metric is a statistic that quantifies the degree to which the network may be divided into such clearly delineated groups [39, 40]. Newman et al. introduced the modularity function and modularity matrix to avoid the influences of random factors so as to obtain the better divisions of the community structure [12, 13, 41]. The modularity *Q* is the number portion of edges falling within communities minus the expected number portion in an equivalent network with edges placed at random. The modularity *Q* can be expressed as follows [42, 43]:

(6) $$\begin{array}{c} Q=\frac{1}{2m}\underset{ij}{\sum }\left(a_{ij}-\frac{k_{i}k_{j}}{2m}\right)\delta \left(i,j\right), \end{array}$$

where *δ*(*i*, *j*)=1 if vertices *i* and *j* belong to the same community or *δ*(*i*, *j*)=0 otherwise. *m*=(∑_*i*_*k*_*i*_)/2 denotes the number of edges in the network, *k*_*i*_ is the degree of the vertex *i*, and *a*_*ij*_ is the weight in the adjacent matrix *A*. Let *B*=*a*_*ij*_ − (*k*_*i*_*k*_*j*_/2*m*), which is called the modularity matrix. Formula (6) can be rewritten in the matrix format as follows:

(7) $$\begin{array}{c} Q=\frac{1}{2m}\text{Trace}\left(X^{T}BX\right), \end{array}$$

where the assignment matrix *X*=(*x*_*ih*_), in which *x*_*ih*_=1 if vertex *i* belongs to the community *h* or *x*_*ih*_=0 otherwise. The function Trace( ) denotes the sum of diagonal elements of a matrix.

A high modularity indicates a better partitioning of the graph. The search for optimal modularity *Q* is an NP-hard problem [44, 45] because the space of possible partitions grows faster than any power of system size.

### 3.2. Conductance [27]

The conductance of a cut is a metric that compares the size of a cut (i.e., the number of edges cut) and the number of edges in either of the two subgraphs induced by that cut. The conductance *ϕ*(*G*) of a graph is the minimum conductance value between all its clusters.

Consider a cut that divides *G* into *k* nonoverlapping clusters *C*_1_, *C*_2_,…, *C*_*k*_. The conductance of any given cluster *ϕ*(*C*_*i*_) is given by the following formula [43, 46]:

(8) $$\begin{array}{c} \text{conductance}=\frac{1}{K}\overset{K}{\underset{k=1}{\sum }}\phi \left(C_{i}\right)=\frac{1}{K}\overset{K}{\underset{k=1}{\sum }}\frac{\sum_{i\in C_{k},j\notin C_{k}}a_{ij}}{\min\left(a\left(C_{i}\right),a\left({\bar{C}}_{i}\right)\right)}\\ =\frac{1}{K}\overset{K}{\underset{k=1}{\sum }}\frac{\sum_{i\in C_{k},j\notin C_{k}}a_{ij}}{\min\left(\sum_{i\in C_{k}}\sum_{j=1}^{N}a_{ij},\sum_{i\notin C_{k}}\sum_{j=1}^{N}a_{ij}\right)}, \end{array}$$

where *K* denotes the number of clusters, *a*_*ij*_ is the weight in the adjacent matrix, *C*_*k*_ represents the *k*-th cluster (*k*=1,2,…, *K*), and *a*(*C*_*i*_) is the number of edges with at least one endpoint in *C*_*i*_. This *ϕ*(*C*_*i*_) represents the cost of one cut that bisects *G* into two vertex sets *C*_*i*_ and ${\bar{C}}_{i}$ (the complement of *C*_*i*_). Since we want to find a number *k* of clusters, we will need *k*−1 cuts to achieve that number. The conductance for the whole clustering is the average value of those *k*−1*ϕ* cuts.

The conductance metric can evaluate how difficultly a random walk is that leaves a cluster [40]. The more difficultly a random walk leaves a cluster is, the more compact cluster is. A low conductance indicates a better partitioning of the graph. The conductance metric usually ranges from 0 to 1, while 0 is the optimal score, which means that each cluster corresponds to a maximal strongly connected component of the network.

## 4. Dataset and Preprocessing

### 4.1. Dataset

A network is a mathematical representation of a real-world complex system and is determined by a collection of nodes (vertices) and links (edges) between pairs of nodes. Brain connectivity datasets comprise networks of brain regions connected by anatomical tracts or by functional associations. Nodes in brain networks usually represent ROIs, while links represent anatomical, functional, or effective connection [40]. A connectivity matrix (CM) is used to store the connectivity strength between all pairs of ROIs in a brain network [47].

The Autism dataset [6] collected 175 individuals with autism spectrum disorder (ASD) and typically developing (TD) ones, which were acquired from 79 resting-state functional MRI (rsfMRI: 42 ASD and 37 TD) brain networks and 94 diffusion tensor imaging (DTI: 51 ASD and 43 TD) brain networks. The dataset can be obtained from the UCLA multimodal connectivity database (http://umcd.humanconnectomeproject.org) [47]. Each rsfMRI imaging is composed of a 264 × 264 connectivity matrix (CM), in which each value denotes the *z*-transformed Pearson correlation coefficient (PCC) [6].

In this study, 79 rsfMRI brain networks (42 ASD and 37 TD) are utilized to test the proposed algorithm.

### 4.2. Data Preprocessing

In this study, we conduct the following preprocessing steps for the above dataset:

1. Reverse *z*-transformation is performed on the original CM to acquire the PCC connectivity matrix (PCM) according to the following formula:

(9) $$\begin{array}{c} x^{\prime }=\frac{e^{2x}-1}{e^{2x}+1}=1-\frac{2}{e^{2x}+1}, \end{array}$$

  where *x* ∈ CM and *x*′ ∈ PCM denote the original and new values, respectively.
(2) The negative data in the PCM signify that the correlation among the vertices is negative correlation. In this study, these negative elements are taken as 0 to get rid of negative correlation.
  After conducting the above two steps, all data in the PCM are in [0, 1], and the PCM turns into a symmetric and nonnegative matrix.
(3) To eliminate data noise, this study adopts the thresholding method to remove all edges with the weight less than a specific value *θ*. Namely, if *x* < *θ*, then *x*=0. In the later numerical experiment, *θ* = 0.2.

## 5. The Proposed Algorithm

In this study, we propose a novel algorithm for finding community modules of brain networks by integrating PSO with the uniform design (abbreviated as UPSO). Its coding and detailed steps are described as follows.

### 5.1. Coding

A brain network *G* can be represented as *G*=(*V*_*G*_, *E*_*G*_), where *V*_*G*_={*v*_1_, *v*_2_,…, *v*_*N*_} is a set of *N*=|*V*_*G*_| vertices and *E*_*G*_={(*v*_*i*_, *v*_*j*_) | *v*_*i*_, *v*_*j*_ ∈ *V*_*G*_} is a set of *M*=|*E*_*G*_| weighted edges (arcs) among *N* vertices. The adjacent matrix of *G* is expressed as *A*=(*a*_*ij*_)_*N*×*N*_, where *a*_*ij*_ denotes the weight between vertices *i* and *j*. From the above-mentioned dataset and data processing, we can see that *A* is a symmetric and nonnegative matrix. The number of community modules and centroid of a community module are denoted by *K* and *CC*_*k*_=(*cc*_*k*1_,…, *cc*_*kN*_),  where *k*=1,2,…, *K*, respectively. In PSO, the position coding *x*_*i*_ of a particle is expressed as

(10) $$\begin{array}{c} x_{i}=\left(CC_{1}^{i},CC_{2}^{i},\ldots ,CC_{K}^{i}\right)=\left(cc_{11}^{i},\ldots ,cc_{1N}^{i},cc_{21}^{i},\ldots ,cc_{2N}^{i},\ldots ,cc_{K1}^{i},\ldots ,c_{KN}^{i}\right), \end{array}$$

where *x*_*i*_ is a *K∗N*-dimensional row vector and *i*=1,…, *N*_pop_ (the population size in PSO).

### 5.2. Detailed Steps

**Figure 1:**
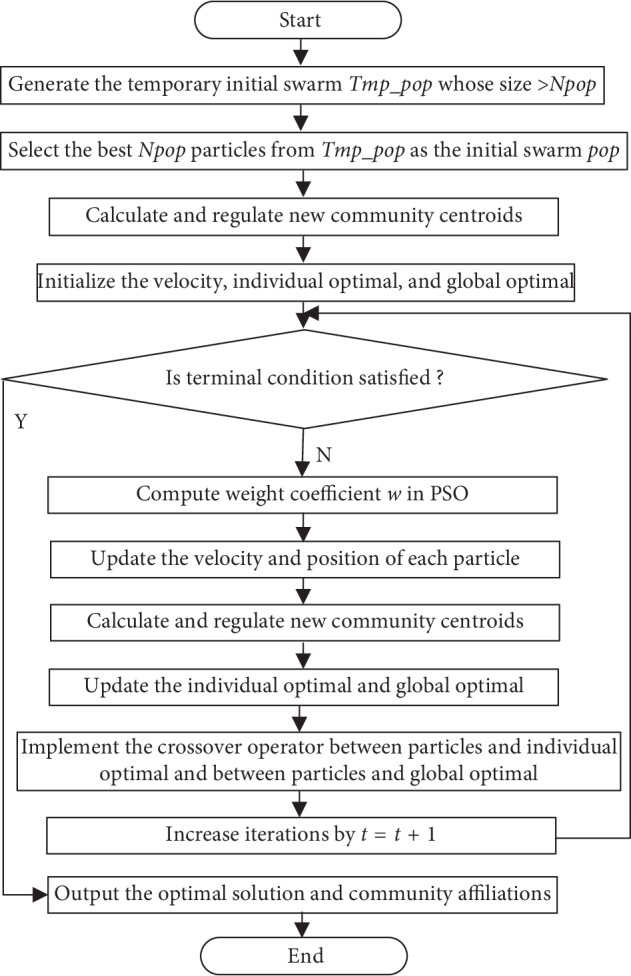
Flow chart of the proposed algorithm.

The proposed algorithm UPSO utilizes the uniform design to obtain the sampled points scattered evenly over the solution space. The initial method based on the uniform design can generate a group of suitable initial particles scattered evenly over the solution space. The crossover operator based on the uniform design can acquire the offspring scattered uniformly over the space spanned by two crossover parents. UPSO iteratively tries to improve a candidate solution in terms of modularity. It integrates the uniform design and PSO to find community modules of brain networks. It can not only obviate the shortcoming of premature convergence in PSO but also acquire the solutions scattered evenly over the solution space. It can find out community modules from brain networks without knowing the number of community modules. Its flow chart is illustrated in Figure 1.

The detailed steps of the proposed algorithm UPSO are described as follows.

**Step 1 .**

(generating a temporary initial swarm). The following operations are performed one after another:

1. Let *K* = *K* + 1, where *K* denotes the number of community modules, and its minimal and maximal values are, respectively, 1 and *N* (the number of vertices). *K* = 1 to *N* is to acquire the fittest number of community modules.
2. According to the swarm size *N*_pop_, the number of subintervals *S* and the swarm size of a subinterval *Q*_0_ are determined such that *S* × *Q*_0_ ≥ *N*_pop_, where *S* can be taken as 2, or 2^2^, or 2^3^, etc.; *Q*_0_ is one of the prime numbers in the first column of Table 1. Here, any combination satisfying *S* × *Q*_0_ ≥ *N*_pop_ can be chosen.
3. The generation algorithm of initial population based on the uniform design described in reference [28] is implemented to generate a temporary initial swarm *Tmp_pop* in terms of *K*, in which each element *x*_*i*_ contains *K* community centroids.

**Step 2 .**

(calculating the fitness of the temporary initial swarm). For each particle *p*_*i*_ in *Tmp_pop*, the following operations are performed in sequence:

1. *K* community centroids *CC*_*k*_ are separated from *x*_*i*_. For each element *a*_*ij*_ in the adjacent matrix *A* described in Section 5.1, the distances are calculated between *a*_*ij*_ and each *CC*_*k*_.
2. Each vertex is assigned to the closest community *C*_*k*_ to obtain its community affiliation *IDX*_*i*_ and *K* community modules.
3. The modularity *Q* of *K* community modules is calculated using formula (6) or (7) in terms of *IDX*_*i*_, and it is taken as the fitness *f*(*x*_*i*_) of *x*_*i*_.

**Step 3 .**

(generating the initial swarm from the temporary initial swarm). According to the acquired fitness of each particle in the temporary initial swarm, the best *N*_pop_ ones of the *Q*_0_*∗S* particles are selected as the initial swarm pop.

**Step 4 .**

(regulating each community module). For each particle position *x*_*i*_ in pop, the following operations are performed in sequence.

The centroid of the community *C*_*k*_ is updated according to the following formula:

(11) $$\begin{array}{c} \bar{CC_{k}}=\frac{1}{n_{k}}\underset{a_{i}\in C_{k}}{\sum }a_{i}, \end{array}$$

where *a*_*i*_=(*a*_*i*1_, *a*_*i*2_,…, *a*_*iN*_), *i*=1,2,…, *N*  and  *N*  is  thenumber  of  vertices;  *k*=1,2,…, *K*; *n*_*k*_ is the number of vertices which belong to the community *C*_*k*_; and $\bar{CC_{k}}$ is the new community centroid of *C*_*k*_.

*K* new community centroids $\left(\bar{CC_{1}},\bar{CC_{2}},\ldots ,\bar{CC_{K}}\right)$ form a new position, marked as *KC*_*i*_, whose fitness and community affiliation are *f*(*KC*_*i*_) and *KC*_*IDX*_*i*_, respectively.

If *f*(*KC*_*i*_) > *f*(*x*_*i*_), then *x*_*i*_ = *KC*_*i*_, *f*(*x*_*i*_)=*f*(*KC*_*i*_), and *IDX*_*i*_ = *KC*_*IDX*_*i*_.

**Step 5 .**

(initializing the velocity *s*_*i*_, individual optimal *P*_best_*i*__, and global optimal *G*_best_). The velocity *s*_*i*_ and individual optimal *P*_best_*i*__ of the particle *p*_*i*_ are initialized as its position *x*_*i*_, and the fitness of *P*_best_*i*__ is set as *f*(*P*_best_*i*__)=*f*(*x*_*i*_). The maximal value in all *P*_best_*i*__ is taken as the global optimal *G*_best_, which stores the best *x*_*i*_ and *Q* of the swarm. The community affiliation *G*_best_ is stored into *IDX*.

**Step 6 .**

(increasing iterations and judging terminal conditions). Let *t* = *t* + 1, then judge whether terminal conditions are satisfied or not, where *t* denotes the *t*-th iteration and its initial value is 0. If *K* is known, and any of the terminal conditions is satisfied, the algorithm terminates and outputs the optimal solution and its community affiliation; otherwise, the algorithm moves to Step 7. Terminal conditions are described in Section 6.1.

**Step 7 .**

(computing the weight coefficient *w* in PSO). The weight coefficient *w* in PSO utilizes a linear decreasing strategy [48, 49] indicated in the following formula:

(12) $$\begin{array}{c} w^{\left(k\right)}=\frac{w_{\max}-t\left(w_{\max}-w_{\min}\right)}{t_{\max}}, \end{array}$$

where *w*_max_ and *w*_min_ are the maximal and minimal values of *w* and *t*_max_ is the maximal number of iterations. In the later numerical experiment, *w*_min_=0.1 and *w*_max_=1.

**Step 8 .**

(updating the velocity and position of each particle). To guide the moving trajectory of a particle by *KC*_*i*_, formula (1) is modified into the following formula:

(13) $$\begin{array}{c} s_{i}\left(t+1\right)=\omega \cdot s_{i}\left(t\right)+c_{1}\cdot r_{1}\left(P_{{\text{best}}_{i}}\left(t\right)-x_{i}\left(t\right)\right)+c_{2}\cdot r_{2}\left(G_{\text{best}}\left(t\right)-x_{i}\left(t\right)\right)+c_{3}\cdot r_{3}\left(KC_{i}\left(t\right)-x_{i}\left(t\right)\right). \end{array}$$

The velocity and position of each particle in the pop are updated in terms of formulas (13) and (2), respectively.

**Step 9 .**

(calculating the fitness and regulating each community module). The fitness of each particle in the pop is calculated according to the operations in Step 2, and Step 4 is implemented to regulate each community module.

**Step 10 .**

(updating *P*_best_*i*__, *G*_best_, and *IDX*). For each particle in the pop, if *f*(*x*_*i*_) > *f*(*P*_best_*i*__), then *P*_best_*i*__=*x*_*i*_ and *f*(*P*_best_*i*__)=*f*(*x*_*i*_).

If *f*(*x*_*i*_) > *f*(*G*_best_), then *G*_best_=*x*_*i*_, *f*(*G*_best_)=*f*(*x*_*i*_), and *IDX* = *IDX*_*i*_.

**Step 11 .**

(implementing the crossover operator based on the uniform design). For each particle in the pop, the following operations are performed in sequence:

1. The crossover operator based on the uniform design is implemented on *x*_*i*_ and *P*_best_*i*__ to acquire the *Q*_1_ offspring scattered uniformly over the space spanned by them and also on *x*_*i*_ and *G*_best_ to acquire another *Q*_1_ offspring.
2. The fitness of the 2*∗Q*_1_ offspring is calculated, and the best one of them is marked as *O*_best_. The fitness and community affiliation of *O*_best_ are expressed as *f*(*O*_best_) and *I*  *DX*_*O*_best__, respectively.
3. If *f*(*O*_best_) > *f*(*P*_best_*i*__), then *x*_*i*_ = *O*_best_, *f*(*x*_*i*_)=*f*(*O*_best_), *P*_best_*i*__=*O*_best_, and *f*(*P*_best_*i*__)=*f*(*O*_best_).
4. If *f*(*O*_best_) > *f*(*G*_best_), then *f*(*G*_best_)=*f*(*O*_best_), *G*_best_=*O*_best_, and *I*  *DX*=*I*  *DX*_*O*_best__.

**Step 12 .**

The algorithm is returned to Step 6.

**Step 13 .**

If *K* < *N*, the best *Q* and community affiliation *IDX* are saved and then the algorithm returns to Step 1; otherwise, the algorithm outputs the optimal solution *G*_best_, the community affiliation *IDX*, and the fittest *K*.

## 6. Numerical Results

In this study, we select four competing community detection algorithms to compare the performances of UPSO. They include the spectral clustering [50], FastQ [14], Danon et al. [15], and Louvain [16] algorithms. FastQ, Danon, and Louvain algorithms are three commonly used community detection methods. Among five algorithms, UPSO and the spectral clustering are stochastic search algorithms, while FastQ, Danon, and Louvain algorithms are deterministic search algorithms.

The parameter values of UPSO and the numerical results obtained by UPSO and four competing algorithms are described as follows.

### 6.1. Parameter Values

In this study, the parameters of UPSO are described as follows.

#### 6.1.1. Parameters for PSO

The minimal and maximal inertia weight coefficients are *w*_min_=0.1 and *w*_max_=1 (the recommended values in PSO); the acceleration coefficients *c*_1_, *c*_2_, and *c*_3_ are all equal to 2 (the recommended values in PSO); the population size *N*pop = 100; the maximal number of iterations *t*_max_ = 100.

#### 6.1.2. Parameters for the Uniform Design

As the above-mentioned each rsfMRI imaging is a 264 × 264 CM, we set the number of subintervals *S* as 4 (*S* can be 2^1^, 2^2^, 2^3^, ......); the number of sample points or the swarm size of each subinterval *Q*_0_ is set as 31 because *Q*_0_ can be any values in Table 1 and the product of *Q*_0_ and *S* must be larger than the population size *N*_pop_, namely, (*Q*_0_*∗S*=31*∗*4=124) > (*N*_pop_ = 100). The parameter *Q*_1_ is set as 5 in order to only generate 5 offsprings in uniform cross to decrease time consumption.

#### 6.1.3. Terminal Conditions

1. The number of iterations *t* > *t*_max_
2. The number of fitness remains unchanged, *t*_no_, and is larger than or equal to 30% of *t*_max_

When any of the above two terminal conditions is satisfied, the algorithm terminates.

It is worth noting that the above parameter values are not fixed and can be changed according to different datasets. The above parameter values are only one of the suitable values, and they do not need to be fine tuned.

As the spectral clustering needs to preestimate the number of community modules, it uses the identical number of community modules to UPSO. FastQ, Danon, and Louvain algorithms do not necessarily need to estimate the number of community modules; therefore, they use their default parameters.

### 6.2. Results

#### 6.2.1. Comparisons of Evaluation Metrics

All the 79 rsfMRI brain networks are utilized to test the performance of five algorithms. Five algorithms independently performed 20 runs to compare their average values. For stochastic search algorithms, UPSO and the spectral clustering, we also compare their standard deviations (the values in parentheses in Tables 2 and 3). Tables 2 and 3, respectively, show the results of modularity and conductance metrics obtained by five algorithms.

From Table 2, it can be obviously observed that, for all 79 rsfMRI brain networks, the modularity metrics obtained by UPSO are all the best among five algorithms. This fully demonstrates that the proposed algorithm outperforms other four competing algorithms in terms of modularity. The main reasons for UPSO obtaining good results are explained as follows: Firstly, UPSO is a heuristic optimization algorithm, so it can search a good solution as much as possible. Secondly, as UPSO is a swarm intelligent optimization algorithm, it can use all the individuals in a swarm to search the optimal solution, while the other four algorithms can use only one individual. Last but not the least, UPSO can use the uniform design to obtain the solutions scattered evenly over the feasible solution space.

In five algorithms, the gaps of the results obtained by UPSO and Louvain algorithm are much less than those by UPSO and other three algorithms, and even UPSO and Louvain algorithm obtain the identical results for ASD90B and ASD95 brain networks. Thus, the Louvain algorithm is the most competing in the other four algorithms.

We can also see from Table 2 that the standard deviations obtained by UPSO and the spectral clustering are all very small compared to the average values obtained by them. This demonstrates that UPSO and the spectral clustering are both relatively stable in terms of modularity for 79 rsfMRI brain networks. Meanwhile, we can also observe that, for 65 of 79 brain networks, the standard deviations obtained by UPSO are less than or equal to those obtained by the spectral clustering. This demonstrates that UPSO has higher stability than the spectral clustering in terms of modularity.

From Table 3, we can clearly see that UPSO obtains the best conductance metrics for most brain networks, but not for all 79 brain networks. This is because that the evaluation perspectives of two metrics are different. However, UPSO obtains the best conductance metrics for 50 brain networks and accounts for about 63% of 79 brain networks. This manifests that UPSO is superior to other competing algorithms in terms of conductance. Meanwhile, this also demonstrates that UPSO can acquire better conductance metrics while ensuring the best modularity metrics. The number of brain networks in that the spectral clustering, FastQ, Danon, and Louvain algorithms obtained the best conductance metrics is 1, 6, 20, and 2, respectively. We can also clearly observe that the best modularity metrics obtained by UPSO and Louvain algorithm are relatively close, but the number of brain networks in that the Louvain algorithm obtained the best conductance metrics is just 2. This fully demonstrates that UPSO outperforms the Louvain algorithm in terms of conductance.

From Table 3, we can also see that the standard deviations obtained by UPSO and the spectral clustering are all very small compared to the average values obtained by them. This is also similar to data in Table 2. Namely, for 79 rsfMRI brain networks, UPSO and the spectral clustering are relatively stable in terms of both modularity and conductance metrics. Meanwhile, we can also observe that, for 42 of 79 brain networks, the standard deviations obtained by UPSO are less than or equal to those obtained by the spectral clustering. This demonstrates that UPSO has higher stability than the spectral clustering in terms of conductance. This conclusion is also similar to that concluded from Table 2.

#### 6.2.2. Comparisons of Other Perspectives

Besides the above-mentioned comparisons, we also evaluate the performances of UPSO from other perspectives, such as influences of the uniform design, comparisons with other heuristic algorithms, and complexity analysis.

To show the benefit of hybridizing the uniform design in PSO, we modify UPSO by removing the uniform design from UPSO. Namely, the initialization (Steps 1, 2, and 3) uses the random initialization method instead of the generation algorithm of the initial population based on the uniform design, and the crossover operator based on the uniform design (Step 11) is not performed. For brevity, the modified algorithm is called PSO. We compare the modularity metrics obtained by UPSO and PSO. The results are shown in Table 4.

To verify the performance of UPSO, we also compare it with ABC (artificial bee colony). Similar to PSO, ABC is also a heuristic algorithm. Table 4 also shows the results obtained by ABC.

**Figure 2:**
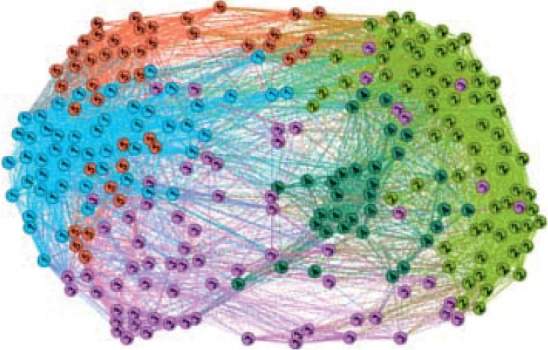
Community plot of TD86C.

**Figure 3:**
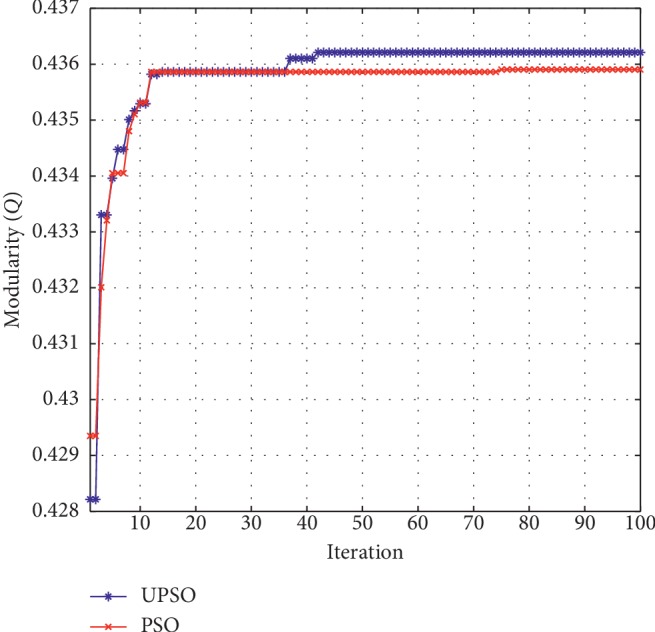
Modularity changing plot of ASD104.

From Table 4, we can clearly see that, for 67 of 79 brain networks, the modularity metrics obtained by UPSO are larger than those obtained by PSO. In comparison, there are just 4 brain networks for which the modularity metrics obtained by UPSO are less than those obtained by PSO. This fully demonstrates that the influence of the uniform design on improving the performance of UPSO is significant. Figures 2 and 3 in the next section also obviously illustrate the benefit of the uniform design.

By comparison of the modularity metrics obtained by UPSO and those obtained by ABC, it can be clearly seen from Table 4 that, for 79 brain networks, the modularity metrics of UPSO are all larger than those of ABC. This fully demonstrates that UPSO significantly outperforms ABC in terms of modularity. A comparison of PSO and ABC is the same as the comparison of UPSO and ABC. Namely, for 79 brain networks, the modularity metrics of PSO are all larger than those of ABC. It follows from the above that PSO is also superior to ABC for 79 rsfMRI brain networks even without the uniform design.

By a detailed analysis of the proposed algorithm UPSO, its computational complexity is obtained as follows: if the number of community modules *K* is pregiven or preestimated, the time complexity of UPSO is *O*(*t*_max_*∗N*_pop_); otherwise, the time complexity of UPSO is *O*(*t*_max_*∗N*_pop_*∗N*), where *t*_max_, *N*_pop_, and *N*, respectively, denote the maximal number of iterations, the population size, and the number of vertices in brain networks. Thus, unless it is absolutely necessary, UPSO often uses the pregiven *K* or the same *K* as that of the other methods to decrease its computational complexity.

#### 6.2.3. Representative Brain Networks

According to different cases of the modularity and conductance metrics in Tables 2 and 3, two representative brain networks are chosen to demonstrate the performance of UPSO.

**Figure 4:**
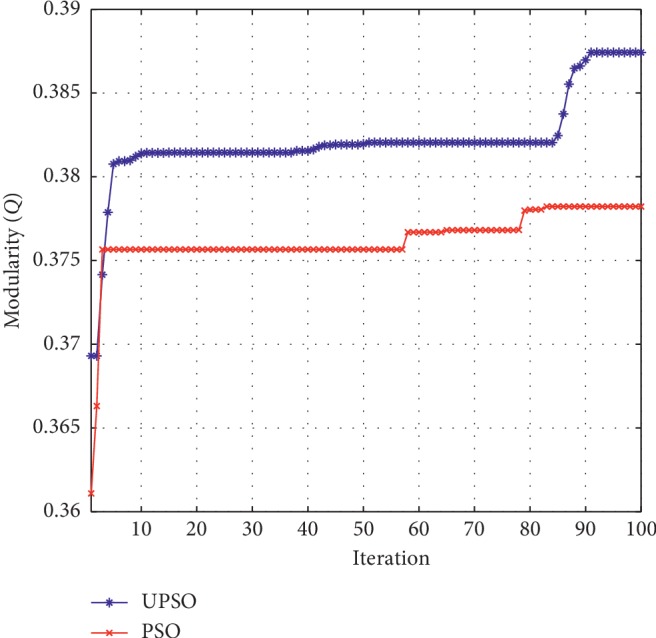
Modularity changing plot of TD86C.

*TD86C Brain Network*. For the TD86C brain network, the best modularity and conductance metrics are both obtained by UPSO.  Figure 4 illustrates the plot of the modularity metrics obtained by UPSO and PSO.

The community plot of the TD86C brain network is illustrated in Figure 2.

From Figure 4, it can be seen that the modularity metrics obtained by UPSO and PSO both converge to a stable state when the number of iterations increases. Meanwhile, we can also clearly see that the plot of UPSO is always above that of PSO after the third iteration. This obviously illustrates that the uniform design plays an important role in improving the performance of UPSO.

**Figure 5:**
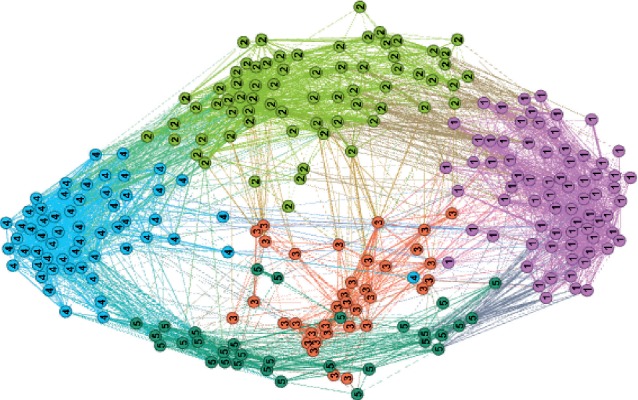
Community plot of ASD104.

*ASD104 Brain Network*. For the ASD104 brain network, the best modularity is obtained by UPSO, while the best conductance metric is obtained by the Danon algorithm.  Figure 3 illustrates the changing process of the modularity metrics obtained by UPSO and PSO with the number of iterations.  Figure 5 illustrates the community plot of ASD104 brain networks.

We can clearly observe from Figure 3 that the plots of UPSO and PSO both go up when the number of iterations increases, which show the processes of searching the optimal solution. However, the plot of UPSO is above or overlapping that of PSO in the whole iterating process. This fully illuminates that the influence of the uniform design is considerable.

## 7. Conclusions and Future Work

In this study, we design a particle swarm algorithm with the uniform design (UPSO) for finding the community modules in brain networks. We conduct UPSO and several competing algorithms on 79 rsfMRI brain networks. The obtained results demonstrate that UPSO can find community modules with maximal modularity and obviously outperforms other competing methods in terms of modularity. The comparison of UPSO and PSO shows that the uniform design plays an important role in improving the performance of UPSO. The comparison of PSO and ABC shows that PSO is superior to ABC for 79 rsfMRI brain networks.

The proposed algorithm UPSO does not apply to very high-dimensional problems because it more likely needs long execution time. To solve the limitations, UPSO can be designed as a parallel algorithm and implemented in the cloud computing platform. In addition, our proposed algorithm is going on for further improvement, such as designing more efficient coding to speed up its converging rate and stability.

## Figures and Tables

**Table 1:** Values of the parameter *σ* for different numbers of factors and different numbers of levels per factor [24, 25].

Number of levels per factor	Number of factors	*σ*
5	2∼4	2

7	2∼6	3

11	2∼10	7

13	2	5
	3	4
	4∼12	6

17	2∼16	10

19	2∼3	8
	4∼18	14

23	2, 13∼14, 20∼22	7
	8∼12	15
	3∼7, 15∼19	17

29	2	12
	3	9
	4∼7	16
	8∼12, 16∼24	8
	13∼15	14
	25∼28	18

31	2, 5∼12, 20∼30	12
	3∼4, 13∼19	22

**Table 2:** Comparisons of the modularity metric.

Dataset	UPSO	Spectral clustering	FastQ	Danon	Louvain
ASD67B	**0.3127** (0.0003)	0.2981 (**0.0000**)	0.2937	0.2930	0.3092
ASD70B	**0.3784** (**0.0013**)	0.3479 (0.0037)	0.3145	0.3341	0.3735
ASD73C	**0.4261** (**0.0010**)	0.3943 (0.0031)	0.3561	0.3767	0.4213
ASD75B	**0.4420** (**0.0000**)	0.3805 (**0.0000**)	0.4174	0.4011	0.4403
ASD76C	**0.4201** (0.0011)	0.4112 (**0.0000**)	0.3135	0.3967	0.4192
ASD82	**0.4300** (**0.0000**)	0.3888 (0.0023)	0.3688	0.3644	0.4281
ASD83B	**0.3527** (**0.0002**)	0.3321 (0.0179)	0.3042	0.3243	0.3522
ASD87B	**0.4551** (**0.0001**)	0.4432 (0.0003)	0.4295	0.4347	0.4536
ASD90B	**0.3919** (0.0025)	0.3699 (**0.0000**)	0.3320	0.3555	**0.3919**
ASD91B	**0.3522** (**0.0020**)	0.3152 (0.0040)0)	0.3402	0.2994	0.3468
ASD92	**0.3987** (**0.0000**)	0.3663 (0.0198)	0.3657	0.3730	0.3954
ASD93B	**0.4153** (**0.0001**)	0.3720 (0.0038)	0.3692	0.3818	0.4089
ASD95	**0.3972** (**0.0005**)	0.3265 (0.0017)	0.3842	0.3765	**0.3972**
ASD96B	**0.4523** (**0.0000**)	0.4092 (0.0381)	0.3550	0.4339	0.4510
ASD97	**0.4233** (**0.0017**)	0.3909 (0.0048)	0.3703	0.3762	0.4187
ASD99	**0.4336** (**0.0001**)	0.4195 (0.0258)	0.3735	0.4194	0.4332
ASD102	**0.4428** (**0.0000**)	0.4325 (0.0015)	0.3523	0.3482	0.4358
ASD103	**0.4443** (**0.0000**)	0.4259 (0.0185)	0.3768	0.4307	0.4439
ASD104	**0.4362** (**0.0000**)	0.4241 (**0.0000**)	0.3868	0.3809	0.4355
ASD106	**0.4194** (**0.0003**)	0.3513 (0.0197)	0.3989	0.4071	0.4192
ASD108	**0.3997** (**0.0003**)	0.3782 (0.0095)	0.3516	0.3590	0.3885
ASD111	**0.4169** (**0.0000**)	0.4109 (0.0004)	0.3742	0.3769	0.4141
ASD112	**0.4515** (**0.0001**)	0.4112 (0.0031)	0.3964	0.3901	0.4477
ASD113	**0.4104** (0.0016)	0.3764 (**0.0003**)	0.3806	0.3778	0.4039
ASD114	**0.3960** (**0.0010**)	0.3590 (0.0045)	0.3765	0.3766	0.3914
ASD115	**0.3903** (**0.0010**)	0.3311 (0.0025)	0.3624	0.3652	0.3861
ASD116	**0.4095** (0.0008)	0.3835 (**0.0007**)	0.3446	0.3074	0.4031
ASD117	**0.4018** (**0.0034**)	0.3684 (0.0043)	0.3613	0.3469	0.3955
ASD119	**0.4386** (**0.0009**)	0.3638 (0.0190)	0.4262	0.4245	0.4368
ASD120	**0.4214** (**0.0001**)	0.3900 (0.0014)	0.3499	0.3810	0.4199
ASD124	**0.3926** (**0.0020**)	0.3574 (0.0085)	0.3021	0.3021	0.3893
ASD125	**0.3935** (**0.0008**)	0.3774 (0.0116)	0.3347	0.3297	0.3916
ASD127	**0.3955** (**0.0012**)	0.3766 (0.0013)	0.3591	0.3390	0.3903
ASD129	**0.3930** (**0.0003**)	0.3425 (0.0017)	0.3579	0.3187	0.3878
ASD130	**0.4208** (**0.0006**)	0.3975 (0.0156)	0.3877	0.3786	0.4115
ASD131	**0.4232** (**0.0021**)	0.3855 (0.0171)	0.4028	0.3967	0.4191
ASD132	**0.4473** (**0.0005**)	0.4195 (0.0026)	0.3516	0.3648	0.4445
ASD133	**0.4249** (**0.0003**)	0.3841 (0.0205)	0.3818	0.3850	0.4221
ASD134	**0.4018** (**0.0006**)	0.3555 (0.0050)	0.3894	0.3418	0.3988
ASD138	**0.4040** (**0.0001**)	0.3972 (0.0085)	0.3853	0.3942	0.3979
ASD142	**0.4349** (**0.0004**)	0.3867 (0.0011)	0.4060	0.4067	0.4337
ASD143	**0.4027** (**0.0001**)	0.3735 (0.0201)	0.3201	0.3521	0.4014
TD86C	**0.3837** (0.0035)	0.3710 (**0.0005**)	0.3387	0.3179	0.3836
TD100C	**0.3910** (**0.0001**)	0.3843 (0.0046)	0.3208	0.3412	0.3864
TD101B	**0.4257** (**0.0002**)	0.3961 (0.0073)	0.4069	0.3990	0.4239
TD102B	**0.4253** (**0.0001**)	0.3715 (**0.0001**)	0.3741	0.3943	0.4221
TD103B	**0.4369** (**0.0005**)	0.3687 (0.0155)	0.4304	0.4217	0.4363
TD105	**0.4295** (**0.0006**)	0.4222 (0.0014)	0.3922	0.3681	0.4265
TD107B	**0.4301** (0.0002)	0.3820 (**0.0000**)	0.4110	0.4042	0.4287
TD108B	**0.4171** (**0.0001**)	0.3689 (0.0010)	0.3723	0.3768	0.4151
TD111B	**0.3341** (**0.0013**)	0.3164 (0.0029)	0.2916	0.2617	0.3286
TD112B	**0.4207** (**0.0001**)	0.3744 (0.0210)	0.3993	0.4128	0.4169
TD113B	**0.4017** (0.0009)	0.3662 (**0.0000**)	0.3481	0.3413	0.3999
TD114	**0.4068** (0.0010)	0.3895 (**0.0000**)	0.3757	0.3552	0.4065
TD118	**0.4267** (**0.0002**)	0.4154 (0.0077)	0.3608	0.3449	0.4243
TD120	**0.4189** (0.0014)	0.3724 (**0.0008**)	0.3699	0.3601	0.4187
TD121	**0.4676** (0.0007)	0.4346 (**0.0001**)	0.4482	0.4337	0.4670
TD122	**0.4738** (**0.0003**)	0.4367 (0.0010)	0.4589	0.4489	0.4733
TD123	**0.4336** (**0.0011**)	0.3805 (0.0070)	0.4179	0.4004	0.4324
TD124	**0.4498** (0.0001)	0.4114 (**0.0000**)	0.3207	0.4043	0.4495
TD125	**0.4028** (**0.0002**)	0.3902 (0.0005)	0.3472	0.3506	0.3988
TD126	**0.4174** (**0.0007**)	0.3623 (0.0012)	0.4051	0.3736	0.4121
TD128	**0.4458** (0.0017)	0.4294 (**0.0000**)	0.3615	0.4038	0.4427
TD129	**0.4396** (**0.0002**)	0.3975 (0.0029)	0.3745	0.3904	0.4375
TD130B	**0.4074** (0.0003)	0.4010 (**0.0001**)	0.3917	0.3637	0.4022
TD131	**0.4104** (**0.0013**)	0.3729 (0.0020)	0.3949	0.3759	0.4079
TD132	**0.4439** (**0.0002**)	0.4356 (0.0005)	0.4068	0.4204	0.4407
TD133	**0.3555** (**0.0003**)	0.3509 (0.0005)	0.3134	0.3154	0.3533
TD134	**0.4317** (**0.0012**)	0.3649 (0.0309)	0.4124	0.3980	0.4285
TD135	**0.4460** (**0.0007**)	0.3664 (0.0231)	0.4200	0.4178	0.4449
TD136	**0.4434** (**0.0001**)	0.4069 (0.0005)	0.3764	0.3731	0.4432
TD137	**0.4276** (**0.0005**)	0.3835 (0.0029)	0.4161	0.3726	0.4270
TD138B	**0.3911** (**0.0003**)	0.3631 (**0.0003**)	0.3696	0.3547	0.3875
TD139	**0.4377** (**0.0000**)	0.4277 (**0.0000**)	0.4230	0.4205	0.4353
TD140	**0.4151** (**0.0009**)	0.3685 (0.0021)	0.3926	0.3791	0.4143
TD142	**0.4373** (**0.0000**)	0.4216 (0.0042)	0.4114	0.3770	0.4295
TD143	**0.4465** (**0.0001**)	0.4392 (0.0003)	0.4111	0.4107	0.4434
TD144	**0.4073** (**0.0002**)	0.3411 (0.0061)	0.3686	0.3635	0.4082
TD145	**0.4540** (**0.0005**)	0.3809 (0.0011)	0.4336	0.4306	0.4494

**Table 3:** Comparisons of the conductance metric.

Dataset	UPSO	Spectral clustering	FastQ	Danon	Louvain
ASD67B	**0.3479** (0.0057)	0.3642 (**0.0001**)	0.5629	0.5598	0.3793
ASD70B	**0.3720** (**0.0028**)	0.4476 (0.0058)	0.5937	0.4961	0.4213
ASD75B	**0.2210** (**0.0000**)	0.2585 (**0.0000**)	0.3912	0.4342	0.2220
ASD76C	**0.2305** (0.0855)	0.2546 (**0.0000**)	0.6694	0.4284	0.3098
ASD82	**0.3155** (**0.0005**)	0.3536 (0.0013)	0.5886	0.3870	0.3179
ASD83B	**0.4531** (**0.0006**)	0.4640 (0.0194)	0.4942	0.4740	0.4547
ASD90B	**0.3387** (0.0325)	0.4247 (**0.0028**)	0.6393	0.4577	0.4074
ASD91B	**0.3461** (0.0354)	0.4360 (**0.0029**)	0.3510	0.6153	0.4206
ASD92	**0.2726** (**0.0000**)	0.3851 (0.0223)	0.6138	0.2923	0.3628
ASD95	**0.3407** (0.0477)	0.3967 (**0.0011**)	0.5686	0.4978	0.3723
ASD97	**0.2773** (0.0471)	0.3541 (**0.0067**)	0.5375	0.3928	0.3382
ASD102	**0.3095** (**0.0001**)	0.3184 (0.0015)	0.5280	0.5410	0.3169
ASD103	**0.2831** (**0.0003**)	0.2995 (0.0424)	0.3565	0.3708	0.2832
ASD106	**0.3569** (**0.0074**)	0.3912 (0.0251)	0.4396	0.4131	0.3577
ASD108	**0.3510** (**0.0019**)	0.3644 (0.0107)	0.4666	0.4813	0.3876
ASD112	**0.3104** (**0.0001**)	0.3501 (0.0024)	0.4750	0.4884	0.3159
ASD113	**0.3505** (0.0298)	0.3725 (**0.0002**)	0.4801	0.5659	0.3703
ASD115	**0.2821** (**0.0009**)	0.3292 (0.0012)	0.5690	0.3497	0.3770
ASD117	**0.3444** (0.0624)	0.4642 (**0.0036**)	0.6117	0.4721	0.3812
ASD119	**0.2305** (0.0442)	0.4344 (**0.0238**)	0.2436	0.2449	0.3521
ASD120	**0.3495** (**0.0010**)	0.3642 (0.0012)	0.3721	0.4348	0.3534
ASD124	**0.3445** (0.0435)	0.3835 (**0.0104**)	0.6241	0.6151	0.3821
ASD127	**0.2640** (**0.0004**)	0.3739 (0.0006)	0.4904	0.3719	0.3395
ASD129	**0.3648** (**0.0000**)	0.4552 (0.0012)	0.5646	0.3807	0.4096
ASD131	**0.3936** (**0.0200**)	0.4379 (0.0268)	0.5357	0.4347	0.4118
ASD132	**0.2245** (**0.0005**)	0.3295 (0.0020)	0.3921	0.3629	0.3633
ASD142	**0.2243** (**0.0011**)	0.3665 (**0.0011**)	0.2653	0.3971	0.3116
ASD143	**0.3914** (**0.0054**)	0.4562 (0.0267)	0.6157	0.4672	0.4204
TD86C	**0.3516** (0.0570)	0.4259 (**0.0003**)	0.6306	0.5136	0.3625
TD101B	**0.2422** (**0.0004**)	0.4026 (0.0073)	0.4243	0.4286	0.3870
TD103B	**0.2280** (**0.0030**)	0.3739 (0.0223)	0.2292	0.2332	0.3158
TD105	**0.2368** (**0.0007**)	0.2454 (0.0017)	0.4263	0.2948	0.3749
TD107B	**0.3336** (0.0024)	0.3661 (**0.0000**)	0.4618	0.4732	0.3413
TD108B	**0.3522** (**0.0003**)	0.3689 (0.0005)	0.4878	0.5875	0.3687
TD111B	**0.3200** (0.0388)	0.4257 (**0.0032**)	0.6067	0.5787	0.4281
TD112B	**0.2342** (**0.0005**)	0.4137 (0.0225)	0.3951	0.4047	0.3708
TD114	**0.2541** (0.0521)	0.3611 (**0.0000**)	0.3000	0.4558	0.3848
TD118	**0.3600** (**0.0125**)	0.3902 (0.0142)	0.4893	0.5571	0.3777
TD120	**0.2535** (0.0508)	0.3719 (**0.0007**)	0.4958	0.4957	0.3676
TD121	**0.2743** (0.0242)	0.3059 (**0.0001**)	0.3811	0.3871	0.2761
TD122	**0.1898** (0.0007)	0.3160 (**0.0006**)	0.2052	0.3717	0.2880
TD126	**0.2602** (0.0454)	0.3878 (**0.0012**)	0.3411	0.3774	0.3747
TD132	**0.2231** (0.0006)	0.2261 (**0.0002**)	0.2465	0.2384	0.2243
TD133	**0.4000** (**0.0006**)	0.4010 (0.0011)	0.5319	0.5189	0.4017
TD134	**0.2502** (0.0526)	0.3739 (**0.0370**)	0.5126	0.4128	0.2649
TD135	**0.2851** (0.0626)	0.4011 (**0.0309**)	0.4591	0.4843	0.3022
TD140	**0.2708** (**0.0017**)	0.3939 (**0.0017**)	0.4986	0.5153	0.3833
TD142	**0.2309** (**0.0000**)	0.2429 (0.0032)	0.5243	0.2889	0.2453
TD144	**0.3574** (**0.0041**)	0.4481 (0.0102)	0.5736	0.3653	0.4091
TD145	**0.2168** (0.0029)	0.3712 (**0.0008**)	0.4763	0.3503	0.3183
ASD93B	0.3384 (**0.0001**)	0.3761 (0.0023)	0.2957	**0.2837**	0.3544
ASD96B	0.2880 (**0.0000**)	0.3455 (0.0434)	0.5993	**0.2223**	0.2876
ASD99	0.3170 (**0.0004**)	0.3303 (0.0322)	0.2893	**0.2439**	0.3179
ASD104	0.3141 (**0.0000**)	0.3259 (**0.0000**)	0.2855	**0.2854**	0.3151
ASD111	0.3839 (**0.0000**)	0.3891 (0.0004)	0.2866	**0.2853**	0.3809
ASD114	0.3693 (0.0044)	0.3948 (**0.0039**)	0.3634	**0.3576**	0.3751
ASD116	0.3974 (0.0138)	0.4165 (**0.0007**)	0.4772	**0.3825**	0.3943
ASD125	0.3478 (0.0264)	0.4216 (**0.0164**)	0.5709	**0.3181**	0.4066
ASD130	0.3349 (**0.0006**)	0.3507 (0.0174)	0.4471	**0.2749**	0.3944
ASD133	0.3337 (**0.0006**)	0.3599 (0.0248)	0.5303	**0.2722**	0.3385
ASD134	0.3563 (0.0334)	0.4460 (**0.0032**)	0.5267	**0.3155**	0.4194
ASD138	0.3463 (**0.0032**)	0.3511 (0.0152)	0.4444	**0.2719**	0.3347
TD100C	0.3565 (**0.0002**)	0.3601 (0.0044)	0.5727	**0.3244**	0.3606
TD102B	0.3387 (0.0003)	0.3805 (**0.0002**)	0.4720	**0.2715**	0.3414
TD124	0.2949 (0.0003)	0.3319 (**0.0000**)	0.4469	**0.2544**	0.2959
TD125	0.3443 (0.0016)	0.3479 (**0.0004**)	0.5600	**0.3071**	0.4080
TD128	0.3055 (0.0183)	0.3264 (**0.0000**)	0.5013	**0.2585**	0.3645
TD129	0.3146 (**0.0015**)	0.3451 (0.0017)	0.3144	**0.2690**	0.3323
TD136	0.3175 (**0.0002**)	0.3432 (0.0005)	0.3995	**0.2914**	0.3177
TD138B	0.3241 (0.0407)	0.3734 (**0.0002**)	0.3291	**0.1568**	0.3231
TD139	0.3146 (0.0001)	0.3255 (**0.0000**)	**0.2431**	0.5279	0.3200
TD143	0.3040 (**0.0002**)	0.3080 (0.0003)	**0.2403**	0.2491	0.2990
ASD73C	0.3280 (0.0014)	0.3459 (**0.0008**)	**0.3091**	0.4343	0.3503
TD113B	0.2731 (0.0675)	0.3749 (**0.0000**)	**0.1559**	0.1613	0.3938
TD123	0.3128 (**0.0004**)	0.3480 (0.0137)	**0.2448**	0.4228	0.3687
TD130B	0.3272 (0.0011)	0.3301 (**0.0002**)	**0.2646**	0.4517	0.3145
ASD87B	0.3111 (0.0010)	**0.2148** (**0.0000**)	0.2325	0.2359	0.2958
TD131	0.3509 (0.0242)	0.3776 (**0.0010**)	0.4897	0.5127	**0.3437**
TD137	0.3402 (**0.0000**)	0.3721 (0.0019)	0.4255	0.4057	**0.3363**

**Table 4:** Comparisons of UPSO, PSO, and ABC.

Dataset	UPSO	PSO	ABC	Dataset	UPSO	PSO	ABC
ASD67B	**0.3127**	0.3121	0.2996	ASD142	**0.4349**	0.4343	0.4281
ASD70B	**0.3784**	0.3759	0.3646	ASD143	**0.4027**	**0.4027**	0.3863
ASD73C	**0.4261**	0.4262	0.4102	TD86C	**0.3837**	0.3824	0.3743
ASD75B	**0.4420**	0.4416	0.4338	TD100C	**0.3910**	0.3909	0.3773
ASD76C	**0.4201**	0.4182	0.4118	TD101B	**0.4257**	0.4200	0.4136
ASD82	**0.4300**	0.4296	0.4192	TD102B	**0.4253**	0.4251	0.4132
ASD83B	0.3527	**0.3555**	0.3341	TD103B	**0.4369**	0.4342	0.4299
ASD87B	0.4551	**0.4552**	0.4475	TD105	**0.4295**	0.4291	0.4174
ASD90B	**0.3919**	0.3910	0.3796	TD107B	**0.4301**	0.4300	0.4199
ASD91B	**0.3522**	0.3506	0.3374	TD108B	**0.4171**	0.4139	0.4038
ASD92	**0.3987**	**0.3987**	0.3835	TD111B	**0.3341**	0.3333	0.3164
ASD93B	**0.4153**	0.4139	0.4036	TD112B	**0.4207**	0.4166	0.4124
ASD95	**0.3972**	0.3968	0.3877	TD113B	**0.4017**	0.4016	0.3898
ASD96B	**0.4523**	**0.4523**	0.4447	TD114	**0.4068**	0.4061	0.3917
ASD97	**0.4233**	0.4220	0.4151	TD118	**0.4267**	**0.4267**	0.4147
ASD99	**0.4336**	0.4335	0.4214	TD120	**0.4189**	0.4150	0.4072
ASD102	**0.4428**	0.4427	0.4324	TD121	**0.4676**	**0.4677**	0.4578
ASD103	**0.4443**	**0.4443**	0.4338	TD122	**0.4738**	0.4733	0.4689
ASD104	**0.4362**	0.4360	0.4253	TD123	**0.4336**	0.4284	0.4226
ASD106	**0.4194**	0.4183	0.4092	TD124	**0.4498**	0.4497	0.4403
ASD108	**0.3997**	0.3996	0.3842	TD125	**0.4028**	0.4026	0.3860
ASD111	**0.4169**	0.4096	0.4039	TD126	**0.4174**	0.4172	0.4064
ASD112	**0.4515**	0.4514	0.4392	TD128	**0.4458**	0.4398	0.4371
ASD113	**0.4104**	0.4102	0.3982	TD129	**0.4396**	0.4395	0.4275
ASD114	**0.3960**	0.3945	0.3836	TD130B	**0.4074**	**0.4074**	0.3950
ASD115	**0.3903**	0.3899	0.3789	TD131	**0.4104**	0.4071	0.4009
ASD116	0.4095	**0.4098**	0.3975	TD132	**0.4439**	0.4438	0.4378
ASD117	**0.4018**	0.4009	0.3907	TD133	**0.3555**	0.3553	0.3423
ASD119	**0.4386**	0.4355	0.4307	TD134	**0.4317**	0.4282	0.4244
ASD120	**0.4214**	0.4212	0.4093	TD135	**0.4460**	0.4426	0.4404
ASD124	**0.3926**	0.3893	0.3793	TD136	**0.4434**	0.4432	0.4304
ASD125	**0.3935**	0.3934	0.3765	TD137	**0.4276**	**0.4276**	0.4185
ASD127	**0.3955**	0.3939	0.3832	TD138B	**0.3911**	0.3903	0.3811
ASD129	**0.3930**	0.3920	0.3801	TD139	**0.4377**	0.4376	0.4254
ASD130	**0.4208**	0.4188	0.4039	TD140	**0.4151**	0.4128	0.4033
ASD131	**0.4232**	0.4227	0.4051	TD142	**0.4373**	**0.4373**	0.4296
ASD132	**0.4473**	0.4465	0.4407	TD143	**0.4465**	0.4464	0.4359
ASD133	**0.4249**	0.4248	0.4117	TD144	**0.4073**	0.4072	0.3937
ASD134	**0.4018**	0.3989	0.3839	TD145	**0.4540**	0.4496	0.4486
ASD138	**0.4040**	0.4038	0.3942				

## Data Availability

The data we used can be publicly available at http://umcd.humanconnectomeproject.org.
